# Week 48 results of a Phase IV trial of etravirine with antiretrovirals other than darunavir/ritonavir in HIV-1-infected treatment-experienced adults

**DOI:** 10.7448/IAS.17.4.19783

**Published:** 2014-11-02

**Authors:** Eduardo Arathoon, Asad Bhorat, Rodica Silaghi, Herta Crauwels, Ludo Lavreys, Lotke Tambuyzer, Simon Vanveggel, Magda Opsomer

**Affiliations:** 1 Clinica Familiar Luis Angel Garcia, Guatemala City, Guatemala; 2 Soweto Clinical Trials Centre, Johannesburg, South Africa; 3 Infectious Diseases Hospital, Brasov, Romania; 4 Janssen Infectious Diseases BVBA, Beerse, Belgium

## Abstract

**Introduction:**

In DUET, etravirine (ETR) 200 mg bid had durable efficacy and a favourable safety profile versus placebo, both arms with an optimised background regimen (BR) including darunavir/ritonavir (DRV/r). TMC125IFD3002 (VIOLIN; NCT01422330) investigated ETR plus ARVs other than DRV/r.

**Materials and Methods:**

This was a 48 week, Phase IV, open-label, single-arm, multicentre study. HIV-1-infected treatment-experienced adult patients on=8 weeks ARV therapy prior to screening, switching either for virologic failure (VF) (viral load [VL] =500 c/mL) or regimen simplification/AEs (RS/AE) (VL<50 c/mL), received active ETR 200 mg bid with an investigator-selected BR of =1 active ARVs, but excluding DRV/r or NRTIs only. The primary objective was to evaluate safety, tolerability and pharmacokinetics (PK).

**Results:**

Of 211 treated patients, 55% were female, 61% black/African American. 155 patients (73%) had baseline (BL) VL=50 c/mL versus 56 (27%) with BL VL<50 c/mL. Between these two latter subgroups, median BL VL was 4.42 versus 1.28 log_10_ c/mL and CD4+ count 238 versus 410.5 cells/mm^3^. Overall, 96% previously used <2 NNRTIs and 99% used=5 PIs; median number of BL NNRTI RAMs was 2, PI RAMs 5 and NRTI RAMs 1. Overall, most common BR ARVs were PIs (83%), mostly lopinavir/r (62%) and mostly used alone (20%) or with 1 or 2 NRTIs (61%). Raltegravir was used in 9% of patients. Most frequent AEs (any cause/grade) were diarrhoea (17%) and URTI (8%). Incidence of grade 3–4 AEs was 13%, serious AEs 5% (no rashes; none ETR related), AEs leading to discontinuation 4%, AEs possibly related to ETR 23% and AEs of interest: rash (any type) 4%, hepatic 6% and neuropsychiatric 3%. At week 48, VF and RS/AE virologic responses (% patients with VL<50 c/mL; FDA Snapshot) were: 48% (74/155) and 75% (42/56), respectively. VF rates were 42% and 13%; 10% and 13% had no VL data in the week 48 window. The percentage of patients adherent to treatment (assessed based on PK sampling plus ETR pill count) was 47% (69/148) and 57% (30/53), in VF and RS/AE, respectively. Median CD4+ count (NC=F) increases were 0.0 and 24.0 cells/mm^3^. In 29/49 of VFs with genotypic data at failure, ETR RAMs emerging in =5 patients were Y181C, E138A and M230L. The geometric mean ETR AUC_12h_ was 4877 ng.h/mL and C_0h_ 293 ng/mL (N=199).

**Conclusions:**

Results of this study were consistent with those for ETR in other similar populations and support the use of ETR 200 mg bid with a non-DRV/r based BR.

